# Noncompaction and Dilated Cardiomyopathy in a Patient with Schizophrenia

**DOI:** 10.1155/2016/7384264

**Published:** 2016-07-31

**Authors:** Josef Finsterer, Claudia Stöllberger

**Affiliations:** ^1^Krankenanstalt Rudolfstiftung, 1030 Vienna, Austria; ^2^2nd Medical Department with Cardiology and Intensive Care Medicine, Krankenanstalt Rudolfstiftung, 1030 Vienna, Austria

## Abstract

*Objectives.* Psychosis and left ventricular hypertrabeculation (or noncompaction) (LVHT) have not been described in the same patient. Here we report a patient with a long-term history of schizophrenia who was later diagnosed with dilated cardiomyopathy (dCMP) and LVHT.* Case Report.* A 47-year-old Caucasian male developed nondifferentiated schizophrenia at the age of 26 y. Since the age of 33 y he was regularly drinking alcohol. At the age of 47 y he developed heart failure. Transthoracic echocardiography showed an enlarged left ventricle, reduced systolic function, and surprisingly LVHT in the apical segment. Additionally, the left atrium was enlarged, the right ventricular cavities were mildly enlarged, and there were pulmonary hypertension and a small pericardial effusion. Cardiac MRI confirmed the echocardiographic findings. Since coronary angiography was normal, dilated cardiomyopathy was additionally diagnosed. Since he was taking clozapine during years, dilated cardiomyopathy could be due to not only alcohol consumption but also the long-term neuroleptic medication.* Conclusions.* LVHT may be associated with nondifferentiated psychosis. Management of LVHT is challenging in patients with psychosis due to poor compliance and adherence of these patients. Patients with LVHT and psychosis need particular attention since they usually take cardiotoxic drugs for a long time, which may further deteriorate the prognosis of LVHT.

## 1. Introduction

Left ventricular hypertrabeculation (LVHT), also known as left ventricular noncompaction (LVNC), is frequently associated with neuromuscular disorders (NMDs) and chromosomal defects [1]. Some of the NMDs also present with involvement of organs other than the muscle, including the brain or the heart [2]. Cerebral involvement in NMDs can be variegated but may also include psychosis [3, 4]. Psychosis and LVHT, however, have not been described in the same patient. Here we report a patient with a long-term history of schizophrenia who was later diagnosed with dilated cardiomyopathy (dCMP) and LVHT.

## 2. Case Report

The patient is a 47-year-old, HIV-negative, Caucasian male, height 180 cm, weight 81 kg, with a history of nondifferentiated schizophrenia since the age of 26 y. Shortly after onset of psychosis he developed fever and severe headache which resolved spontaneously without therapy or residual deficits. Although he did not undergo CSF investigations, meningitis was retrospectively suspected. Exacerbations of psychosis required recurrent admissions to psychiatric departments at ages 26 y, 28 y, 29 y, 31 y, and 34 y and were successfully treated with neuroleptics. During the first psychotic episode he developed severe pneumonia. Temporarily he was also taking cannabinoids. Additionally, he suffered from chronic alcohol disease since the age of 33 y. His father had a history of questionable Guillain-Barre syndrome (GBS) with complete remission and prostate cancer but no cardiac compromise and no psychiatric disorder.

**Figure 1:**
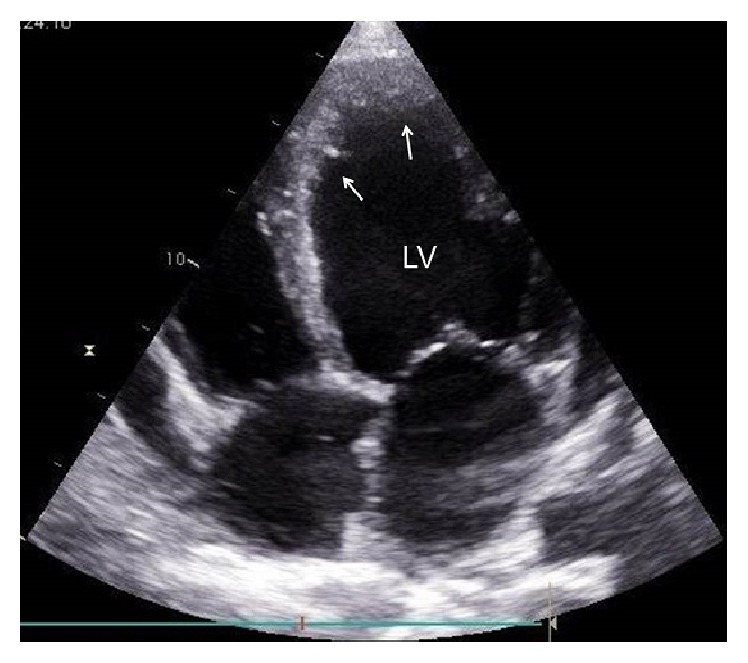
Echocardiographic apical 4-chamber view showing an enlarged left ventricle (LV) with hypertrabeculation/noncompaction affecting the left ventricular apex, the apical septum, and lateral wall (arrows).

**Figure 2:**
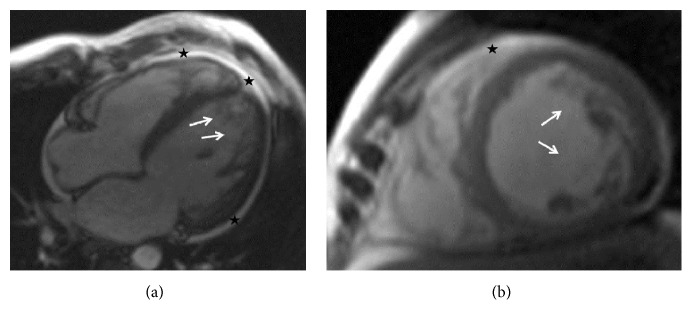
Cardiac MRI, cine mode, showing apical hypertrabeculation (arrows) and circumference pericardial effusion (asterisk) ((a) 4-chamber view, (b) midventricular 2-chamber view).

At the age of 47 y he was hospitalised because of extensive leg edema, weight gain, and resting dyspnoea. X-ray of the lungs showed an enlarged heart and pulmonary congestion grade I. Blood pressure was 110/80 mmHg. Transthoracic echocardiography showed an enlarged left ventricle with an ejection fraction (EF) of 15–20% and surprisingly LVHT in the apical segment (Figure 1). Additionally, the left atrium was enlarged, the right ventricular cavities were mildly enlarged, and there were secondary pulmonary hypertension (55 mmHg) and a small pericardial effusion. Cardiac MRI revealed an enlarged left ventricle, with increased myocardial mass and markedly reduced systolic function and severe diffuse hypokinesia (Figure 2). There was no late enhancement but bilateral enlargement of both atria and a small pericardial effusion. LVHT was confirmed by cardiac MRI (Figure 2). Since coronary angiography was normal, dCMP was diagnosed. ECG showed signs of left ventricular hypertrophy. Blood tests revealed hyperbilirubinemia, hypocalcemia, and occasional hyperCKemia with values up to 236 U/L (*n *<190 U/L). NT-proBNP values were elevated to 2871 ng/L (*n* <84 ng/L). Intravenous diuretic therapy led to a weight loss of 19 kg. A therapy with candesartan and nebivolol was started. Because of severely reduced systolic function and the increased risk of sudden death, a wearable cardioverter (LifeVest®) was recommended but refused by the patient. Since clozapine has an arrhythmogenic effect [5, 6] and a potential to induce cardiomyopathy [7] or myocarditis [8], psychiatrists recommended switching to aripiprazole which the patient refused. Two months after discharge, the patient was in NYHA stage I of heart failure and echocardiography showed only a slight improvement in systolic function with an EF of 30%. He was taking candesartan (20 mg/d), nebivolol (2.5 mg/d), and spironolactone (25 mg/d). Because of low systolic blood pressure, the neurohumoral therapy could not be uptitrated into the target dose so far. He continued refusing a LifeVest or an implantable cardioverter/defibrillator. His last psychiatric medication included clozapine 6.25 mg/d. Clinical neurologic examination was normal and the patient refused work-up for hyperCKemia.

## 3. Discussion

The presented patient is interesting for two aspects. First, the patient had dCMP, LVHT, pulmonary hypertension, and additionally psychosis. Whether there was a causal relation between these four conditions is unknown. No reports about such an association are available from the literature. Whether dCMP is attributable to the chronic alcohol abuse remains speculative. An argument for a causal relation is that dCMP was detected after onset of chronic alcoholism. Strong arguments against a causal relation, however, are that the patient did not present with chemical stigmata of chronic alcoholism, such as hepatopathy or megaloblastic anemia, and that dCMP started 14 y after onset of alcohol abuse. dCMP has been also reported due to clozapine therapy. However, dCMP occurred long after clozapine was started and at a maintenance dosage of 6.25 mg, which makes a causal relation unlikely. Since presumed meningitis was time-related to psychosis it cannot be excluded that psychosis was symptomatic and not endogenous. Arguments in favour of a causal relation between the psychiatric abnormality and LVHT is that LVHT is frequently associated with depression [9] and that the neuromuscular disorder most frequently associated with LVHT may also manifest with psychosis. The combination of psychosis, LVHT, pulmonary hypertension, recurrent CK elevation, dilated CMP, and presumed aseptic meningitis thus suggests a common metabolic defect in this patient.

Second, the patient's father was diagnosed with GBS upon quadriparesis, double vision, and ptosis shortly after a bronchitic infection. However, nerve conduction studies revealed only an axonal lesion with normal velocities in two nerves. CSF investigations were also normal. Despite these atypical findings, he underwent several cycles of plasmapheresis with immunoglobulins with a beneficial effect. At onset of therapy he became delirious. Despite two relapses during hospitalisation, he was released after three weeks without deficits. Since the father did not consent to a follow-up investigation, 25 y after the neuromuscular episode, it remains unclear whether there was a causal link between the previous neuromuscular compromise of the father and the psychiatric and cardiac compromise and hyperCKemia of the son. It also remains unclear whether the father had LVHT or not.

In conclusion, this case shows that LVHT and psychosis may occur together in a single patient, which has not been reported thus far. However, a causal relation between LVHT and psychosis remains speculative. Management of LVHT and hyperCKemia is difficult in patients with psychosis due to poor compliance and adherence of these patients. Patients with LVHT and psychosis need particular attention also because they usually take cardiotoxic drugs for a long time, which may further deteriorate the prognosis of LVHT.
